# Clinical sign and biomarker-based algorithm to identify bacterial pneumonia among outpatients with lower respiratory tract infection in Tanzania

**DOI:** 10.1186/s12879-021-06994-9

**Published:** 2022-01-06

**Authors:** Sarika K. L. Hogendoorn, Loïc Lhopitallier, Melissa Richard-Greenblatt, Estelle Tenisch, Zainab Mbarack, Josephine Samaka, Tarsis Mlaganile, Aline Mamin, Blaise Genton, Laurent Kaiser, Valérie D’Acremont, Kevin C. Kain, Noémie Boillat-Blanco

**Affiliations:** 1 Infectious Diseases Service, University Hospital and University of Lausanne, Lausanne, Switzerland; 2 Tropical Disease Unit, Department of Medicine, Sandra Rotman Centre for Global Health, University Health Network-Toronto General Hospital, University of Toronto, Toronto, Canada; 3 Department of Radiology, University Hospital and University of Lausanne, Lausanne, Switzerland; 4 Mwananyamala Hospital, Dar es Salaam, United Republic of Tanzania; 5 Ifakara Health Institute, Dar es Salaam, United Republic of Tanzania; 6 Division of Infectious Diseases and Center for Emerging Viral Diseases, University of Geneva Hospitals, and Faculty of Medicine, Geneva, Switzerland; 7 Center for Primary Care and Public Health, University of Lausanne, Lausanne, Switzerland; 8 Swiss Tropical and Public Health Institute, University of Basel, Basel, Switzerland

**Keywords:** Bacterial community-acquired pneumonia, Predicting algorithm, Biomarkers, PCT

## Abstract

**Background:**

Inappropriate antibiotics use in lower respiratory tract infections (LRTI) is a major contributor to resistance. We aimed to design an algorithm based on clinical signs and host biomarkers to identify bacterial community-acquired pneumonia (CAP) among patients with LRTI.

**Methods:**

Participants with LRTI were selected in a prospective cohort of febrile (≥ 38 °C) adults presenting to outpatient clinics in Dar es Salaam. Participants underwent chest X-ray, multiplex PCR for respiratory pathogens, and measurements of 13 biomarkers. We evaluated the predictive accuracy of clinical signs and biomarkers using logistic regression and classification and regression tree analysis.

**Results:**

Of 110 patients with LRTI, 17 had bacterial CAP. Procalcitonin (PCT), interleukin-6 (IL-6) and soluble triggering receptor expressed by myeloid cells-1 (sTREM-1) showed an excellent predictive accuracy to identify bacterial CAP (AUROC 0.88, 95%CI 0.78–0.98; 0.84, 0.72–0.99; 0.83, 0.74–0.92, respectively). Combining respiratory rate with PCT or IL-6 significantly improved the model compared to respiratory rate alone (p = 0.006, p = 0.033, respectively). An algorithm with respiratory rate (≥ 32/min) and PCT (≥ 0.25 μg/L) had 94% sensitivity and 82% specificity.

**Conclusions:**

PCT, IL-6 and sTREM-1 had an excellent predictive accuracy in differentiating bacterial CAP from other LRTIs. An algorithm combining respiratory rate and PCT displayed even better performance in this sub-Sahara African setting.

**Supplementary Information:**

The online version contains supplementary material available at 10.1186/s12879-021-06994-9.

## Background

Antimicrobial resistance (AMR) is a growing problem associated with antibiotic use [1, 2]. Most antibiotics are prescribed in outpatient clinics and lower respiratory tract infections (LRTI) account for the majority of unnecessary prescriptions [3, 4].

Community-acquired pneumonia (CAP), usually of bacterial origin, requires antibiotic treatment according to guidelines while other LRTIs such as bronchitis are generally self-resolving [5]. The presence of a new infiltrate on chest X-ray remains the gold standard to decide on antibiotic prescription among patients with LRTIs, even though it has a limited performance and cannot differentiate viral from bacterial aetiologies [6]. Recent studies investigating the causes of CAP using molecular microbiology identified a respiratory virus in a quarter of patients [7]. The proportion of patients with viral CAP is even higher during outbreaks, such as the ongoing SARS-CoV-2 pandemic, highlighting the need for easy-to-perform diagnostic tools to support clinicians in patient management and allow rational antibiotic use.

Biomarkers can support clinical decision-making in patients with LRTI. Studies have evaluated the utility of host immune and endothelial activation biological markers to diagnose pneumonia. Soluble trigger receptor expressed on myeloid cells-1 (sTREM-1), C-reactive protein (CRP), procalcitonin (PCT), Angiopoietin-1 (Angpt-1) and Angiopoietin-2 (Angpt-2) showed promising results in this context [8, 9]. To our knowledge, no study has evaluated host biomarkers to identify adults with bacterial CAP versus other causes of LRTI in sub-Saharan Africa.

In low- and middle-income countries, resources for diagnosing pneumonia are often lacking. In the following study, we hypothesized that an algorithm based on clinical signs and host biomarkers can predict bacterial CAP among patients with LRTIs in outpatient clinics in low resource settings.

## Methods

### Study design and population

This study was nested in a prospective cohort study on fever aetiology conducted between July 2013 and May 2014 in four outpatient clinics (one public hospital and three connected health care facilities) in Dar es Salaam, Tanzania [10]. Consecutive patients (age ≥ 18 years) with fever (tympanic temperature ≥ 38 °C) were included in the cohort if they met inclusion criteria: (1) fever for ≤ 7 days and (2) first consultation for the presenting complaint. Exclusion criteria were: refusal of HIV-1 screening, injury or trauma as the main reason for consultation, delivery within 6 weeks of presentation or hospital admission within 1 month.

In this nested study, we included patients with a clinical LRTI. We defined a clinical LRTI as cough and/or dyspnoea combined with at least one of the following sign or symptoms: tachypnoea (respiratory rate ≥ 20/min), abnormal chest auscultation and/or chest pain. Patients in whom the respiratory tract symptoms were due to another cause than LRTI (such as patients with typhoid fever and bacteremia from another source than the respiratory tract) were excluded. Patients with tuberculosis/fungal infection were excluded as they can be identified by specific microbiological tests and need specific treatment. Of note, tuberculosis was defined as the presence of a positive GeneXpert MTB/RIF in sputum, a positive TB LAM Ag in urine or a chest X-ray suggestive of tuberculosis and the decision by the medical doctor in charge to treat with a full course of treatment.

Patient demographics, co-morbidities, symptoms, as well as vital and other clinical signs were collected at inclusion in the outpatient clinic using a standardised electronic case report form.

Two easy-to-measure bedside clinical scores to identify patients at risk of poor outcome were calculated at inclusion, CRB-65 and quick Sequential Organ Failure Assessment (qSOFA). The van Vugt score [11, 12], a clinical score to predict pneumonia, was also measured at inclusion: one point each for absence of runny nose, presence of dyspnea, presence of crackles or diminished breath by auscultations, temperature ≥ 37.8 °C or heart rate > 100/min. Mortality was assessed by a phone call on day 28. If the patient was unreachable, we called a patient’s relative.

### Microbiological investigations and chest X-ray

All patients had a rapid diagnostic test for HIV-1, dengue, malaria, typhoid as well as blood cultures. A nasopharyngeal swab was collected in all patients at inclusion and stored at − 80 °C until analysis. Retrospectively, a multiplex polymerase chain reaction (PCR) for 11 respiratory bacteria*, Pneumocystis jirovecii* and 21 respiratory viruses was performed on nasopharyngeal swabs (Fast-track DIAGNOSTICS respiratory pathogens 33® (ref FTD-2P.3-64)) in all patients. According to a predefined algorithm [10], selected patients were screened for tuberculosis (GeneXpert MTB/RIF in two sputa, TB LAM Ag in urine), histoplamosis (urinary Histoplasma antigen and Histoplasma IgM in serum) and/or, *Pneumocystis jirovecci* (in induced sputum by immunofluorescence and PCR and in serum by (1,3-β-d-glucan).

All patients with a clinical LRTI had a chest X-ray. Two experienced radiologists read the X-rays and decided on the presence of lung infiltrate suggestive of pneumonia. A third radiologist solved discordant results.

### Definitions

We classified LRTI patients in four groups according to the radiological and microbiological results: group 1 (patients with bacterial CAP: presence of an infiltrate on chest X-ray and positive blood culture and/or nasopharyngeal positive PCR for *Streptococcus pneumoniae*, *Haemophilus influenzae* and/*or Moraxella catarrhalis* (cycle threshold < 35), *Mycoplasma pneumoniae*, *Chlamydia pneumoniae* and/or *Legionella pneumophila* (any positive PCR result) irrespective of the presence of a respiratory virus), group 2 (patients with viral CAP: presence of an infiltrate on chest X-ray and a positive respiratory virus PCR (influenza, picornavirus, RSV, adenovirus, parainfluenza, coronavirus, bocavirus, metapneumovirus and/or enterovirus), group 3 (patients with CAP of unknown origin: presence of an infiltrate on chest X-ray and negative nasopharyngeal PCR for respiratory viruses and bacteria) and group 4 (patients with bronchitis: absence of infiltrate on chest X-ray). We did not consider nasopharyngeal PCR results in bronchitis as these patients do not need antibiotics anyway.

### Quantification of markers of immune and endothelial activation

Plasma samples were collected from all patients at enrolment in the outpatient clinic and stored at − 80 °C within four hours of blood collection. Plasma concentrations of markers of endothelial- and immune activation were analysed using a multiplex Luminex® platform with custom-developed reagents from R&D Systems (Minneapolis, MN) as described [13, 14]. PCT (RayBiotech®, Norcross, GA) and CRP (R&D DuoSet®, Minneapolis, MN) were quantified by enzyme-linked immunosorbent assay.

### Statistical analysis

Differences in characteristics, vital signs, management, outcome and biomarkers between the four groups were evaluated by Kruskal–Wallis or Chi-squared test, as appropriate. Differences between bacterial CAP and patients with other LRTIs were evaluated using Mann Whitney-U, *chi*-squared, or Fisher tests as appropriate. P values were adjusted for multiple comparisons using Bonferroni correction. Using univariate logistic regression, the area under the receiver operating characteristic curve (AUROC) was calculated for all clinical signs and biomarkers to predict bacterial CAP among patients with LRTIs. Variables with excellent predictive value (AUROC ≥ 0.80) were selected for the multivariate analysis [15]. The linearity of the continuous variables with respect to the logit of the dependent variable was assessed via the Box-Tidwell (1962) procedure and by inspecting the partial residuals [16]. Nonlinear variables were transformed for the multivariate logistic regression. A maximum of two variables at a time were tested to avoid overfitting (rule of the thumb of testing one variable per ten events—bacterial CAP). The predictive validity of a multivariate model adding top biomarkers to vital signs was measured using logistic regression, and the predicted probabilities were used to generate AUROC. The multi-variate models were compared using the DeLong method [17].

A classification and regression tree analysis (CRT) was performed with all vital signs and biomarkers with the following settings: minimum of ten cases for parent node and five for child node, pruning to reduce overfitting, and maximum levels for tree depth of three [18]. To ensure safety, misclassification cost for radiological bacterial CAP was set ten times greater than the misclassification cost for other LRTI.

The identified algorithm was tested to predict bacterial CAP versus CAP of other aetiology (viral or unknown).

To evaluate the appropriateness of algorithm recommendation regarding antibiotics, we compared, among patients who received antibiotics during routine care, the characteristics of those in whom antibiotics were recommended by the algorithm to those in whom antibiotics were not recommended using Mann Whitney-U, *chi*-squared, or Fisher tests as appropriate.

All analyses were performed with IBM SPSS version 26 (IBM Corporation, Armonk, New York, USA), STATA software (version 13.1, Stata Corp, College Station, TX, USA) MedCalc version 19.1 and GraphPad Prism 8.

## Results

**Fig. 1:**
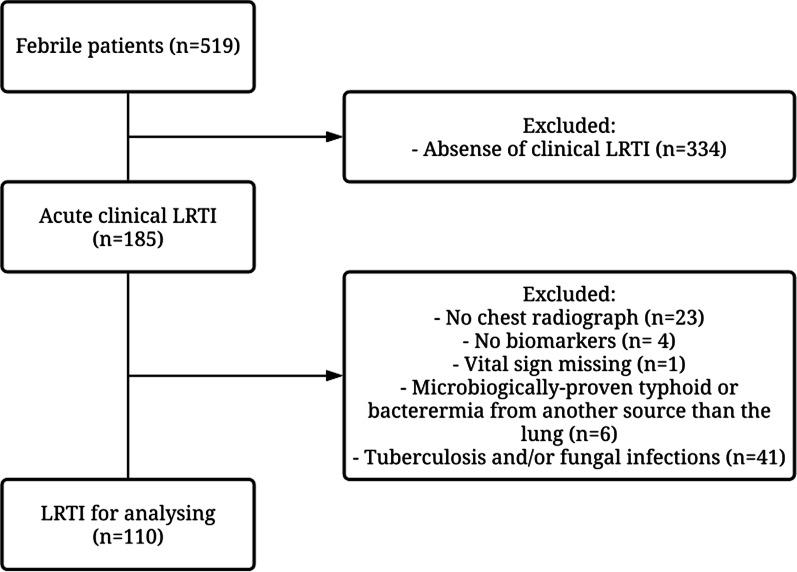
Study flow chart. *LRTI* lower respiratory tract infection

**Fig. 2:**
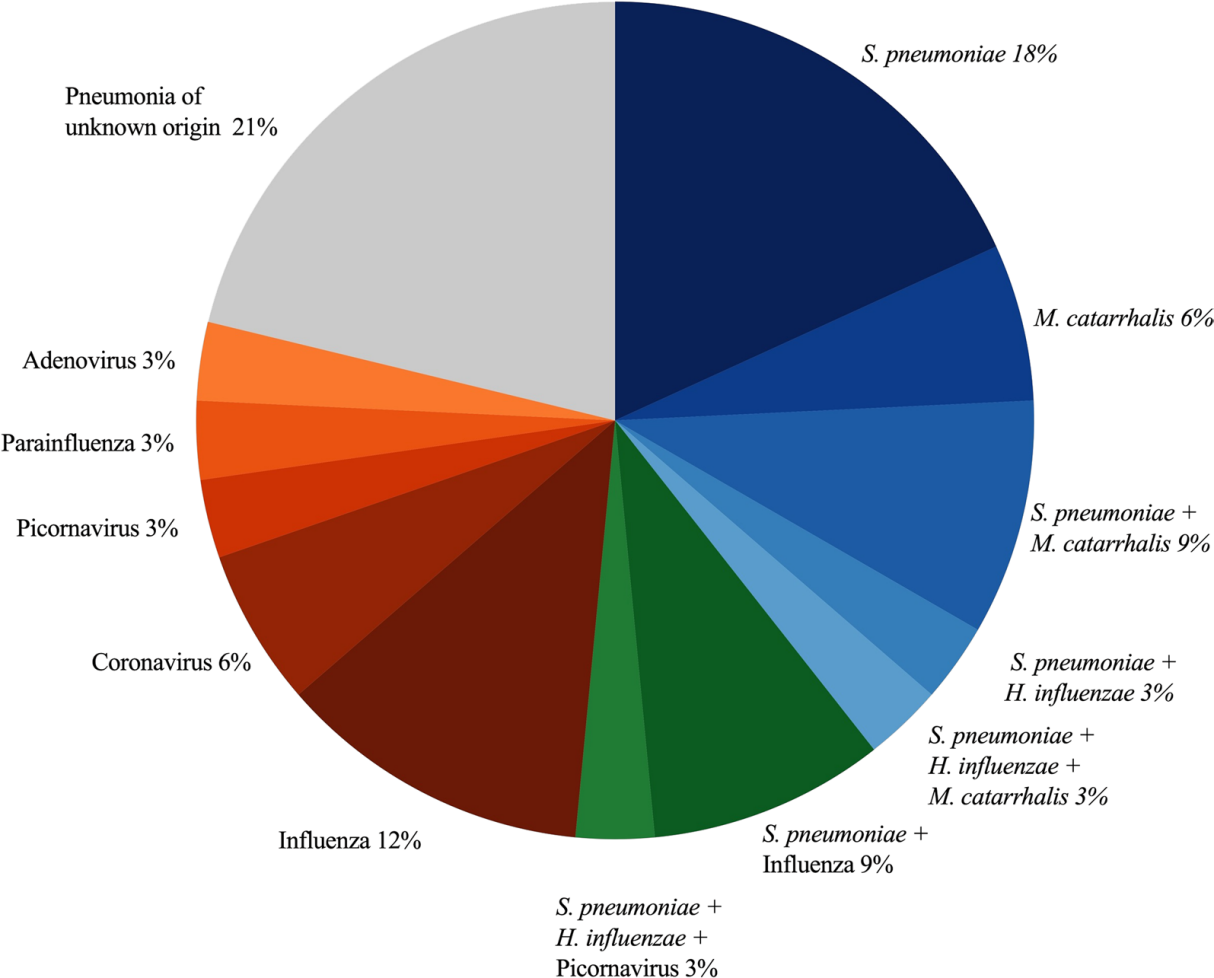
Aetiologies distribution of community-acquired pneumonia (n = 32). Of note, no *Mycoplasma pneumoniae*, *Chlamydia pneumoniae* or *Legionella pneumophila* were identified

Among 519 patients prospectively enrolled in the fever aetiology cohort, 110 patients with a clinical LRTI and no exclusion criteria were included in this study (Fig. 1): 17 in group 1 (bacterial CAP), 8 in group 2 (viral CAP), 7 in group 3 (CAP of unknown origin) and 78 in group 4 (bronchitis). Figure 2 shows the pathogens distribution in patients with CAP (groups 1 to 3). Additional file 1: Tables S1 and S2 shows additional analyses supporting the accuracy of our bacterial CAP (group 1) definition.

### Demographic and clinical characteristics

Table 1 shows the demographic and clinical characteristics of the study population. The median age of the study population was 29 years. Fifty-three percent were female and 33% were HIV-infected. In total, 50% of the patients received an antibiotic and 5% died within 28 days. Patients with bacterial CAP were more likely to be HIV-infected (76% versus 25%; < 0.001), and have an elevated CRB-65 score (29% versus 8%; p = 0.012) compared to those with another LRTI (Additional file 1: Table S3).

**Table 1:** Baseline characteristics, vital signs, management and outcome of patients according to the diagnosis

	All (n = 110)	Bacterial CAP (n = 17)	Viral CAP (n = 8)	CAP of unknown origin (n = 7)	Bronchitis (n = 78)	p-value
Age, years	29 (23–39)	28 (24–35)	31 (29–51)	33 (32–64)	29 (21–38)	0.051
Female sex	58 (53)	8 (47)	3 (38)	2 (29)	45 (58)	0.338
HIV-1infection	36 (33)	13 (76)	4 (50)	1 (14)	18 (23)	**0.000**
Co-infection	22 (20)	2 (12)	1 (13)	1 (14)	18 (23)	0.658
- Malaria	7 (6.4)	1 (5.9)	0 (0)	0 (0)	6 (7.7)	0.739
- Dengue	7 (6.4)	0 (0)	0 (0)	0 (0)	7 (9.0)	0.381
- Other	9 (8.2)^a^	1 (5.9)	1 (13)	1 (14)	6 (7.7)	0.875
Sepsis / septic shock^b^	27 (25)	5 (35)	1 (13)	1 (14)	20 (26)	0.731
*Symptoms and signs*						
Cough	103 (94)	17 (100)	8 (100)	6 (86)	72 (92)	0.446
Dyspnoea	27 (25)	6 (35)	1 (13)	2 (29)	18 (23)	0.606
Chest pain,	25 (23)	4 (24)	3 (38)	2 (29)	16 (21)	0.716
Respiratory rate, /min	25 (23–29)	34 (26–37)	26 (25–34)	24 (23–26)	24 (22–26)	**0.000**
Abnormal auscultation	29 (26)	8 (47)	2 (25)	2 (29)	17 (22)	0.202
Saturation, %	97 (96–98)	95 (93–96)	96 (94–96)	97 (96–98)	97 (96–98)	**0.000**
Systolic BP, mmHg	117 (104–123)	100 (97–107)	119 (109–123)	126 (120–132)	118 (104–124)	**0.000**
Heart rate, /min	108 (92–120)	129 (117–138)	115 (104–120)	98 (82–108)	106 (89–115)	**0.000**
*Pneumonia clinical scores*						
CRB-65 score^c^ ≥ 2	12 (11)	5 (29)	1 (13)	0 (0)	6 (7.7)	0.053
Van Vugt-score^d^, high	68 (62)	16 (94)	6 (75)	4 (57)	42 (54)	**0.016**
*Management and outcome*						
Admission	20 (18)	7 (41)	2 (25)	2 (29)	9 (12)	**0.028**
Antibiotic prescription	55 (50)	16 (94)	7 (88)	4 (57)	28 (36)	**0.000**
28-day mortality	6 (5.5)	2 (12)	1 (13)	0 (0)	3 (3.9)	0.411

Bold values indicate a significant difference between the groups

Data are number (%) of patients or median (interquartile range)

Bacterial CAP: community-acquired pneumonia with a bacterial aetiology detected; viral CAP: community-acquired pneumonia with a viral aetiology detected. CAP of unknown origin: community-acquired pneumonia without microbiological documentation

^a^1 adenovirus gastro-enteritis, 3 urogenital infections, 2 West nile, 1 intraabdominal infection, 2 ricketsioses, 2 gastroenteritis of unknown origin. ^b^Sepsis or septic shock defined as a sofa score of ≥ 2 points. ^c^CRB-65 score defined as one point for each of the following: Glasgow Coma Score < 15, respiratory rate ≥ 30/min, systolic blood pressure < 90 mmHg or diastolic blood pressure ≤ 60 mmHg, age ≥ 65. ^d^Van Vugt score was defined as one point for each of the following: absence of runny nose, presence of dyspnea, presence of crackles or diminished breath by auscultations, temperature ≥ 37.8 °C or heart rate > 100/min. High score was defined as ≥ 3 points

**Fig. 3:**
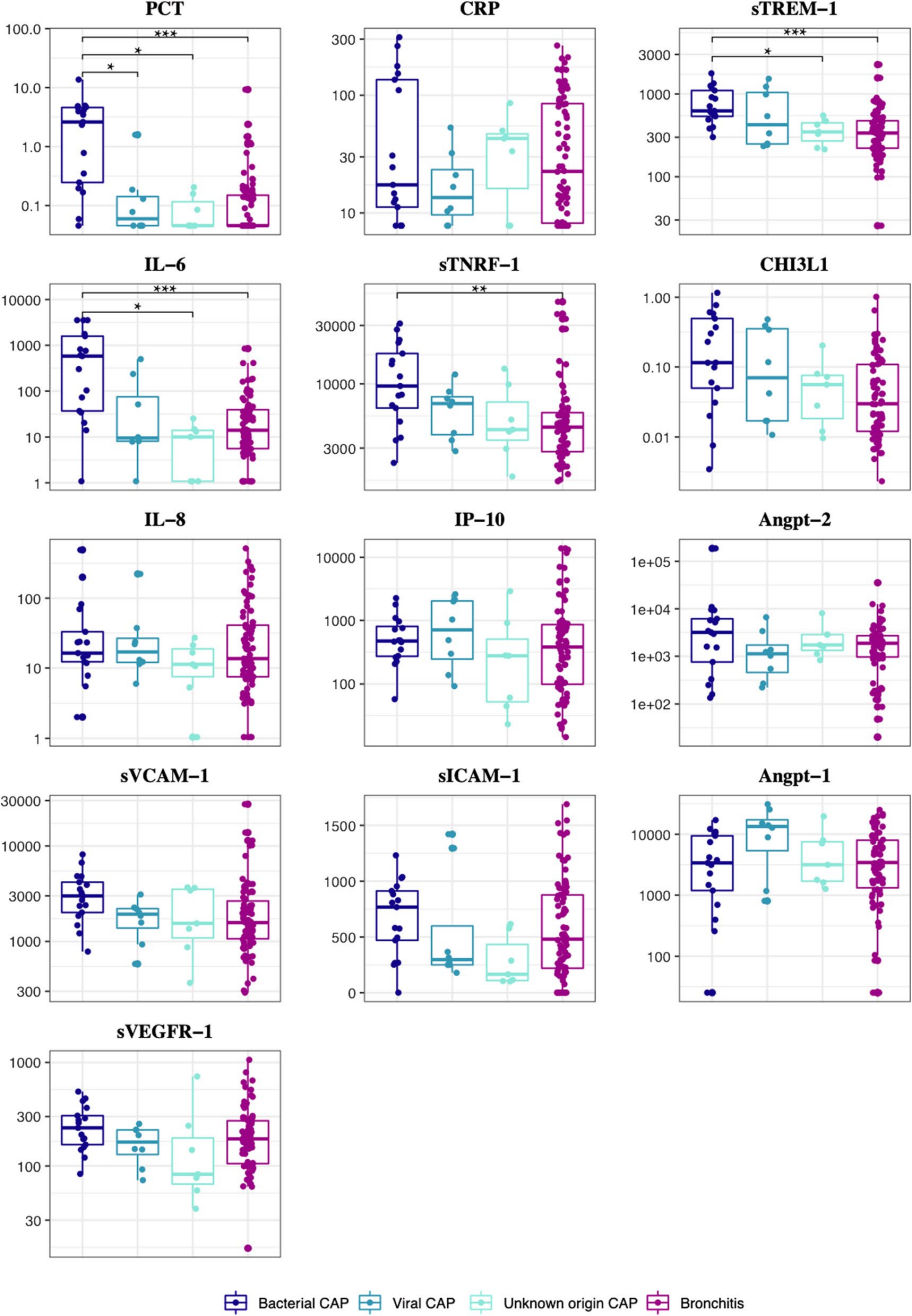
Plasma concentration of immune and endothelial dysfunction markers at clinical presentation according to the diagnosis. Boxplot with median and interquartile range. Concentrations reported in pg/mL except CRP in mg/L. P values were computed using the Wilcoxon-Mann Whitney test and were adjusted for multiple comparisons using Bonferroni method. P * < 0.05; ** < 0.01; *** < 0.001. CAP, community-acquired pneumonia

PCT was significantly higher in patients with bacterial CAP compared to other LRTI groups, while CRP did not show any significant difference. IL-6 and sTREM-1 were significantly higher in patients with bacterial CAP compared to those with CAP of unknown origin and bronchitis (Table 2, Fig. 3).

**Table 2:** Plasma concentration of immune and endothelial dysfunction markers at clinical presentation according to the diagnosis

	All (n = 110)	Bacterial community-acquired pneumonia (n = 17)	Viral community-acquired pneumonia (n = 8)	Unknown origin community-acquired pneumonia (n = 7)	Bronchitis (n = 78)	P value
*Common biomarkers*						
CRP mg/l	22 (8–85)	17 (10–144)	14 (8–29)	43 (8–50)	23 (8–86)	0.661
PCT µg/l	0.09 (0.05–0.21)	2.62 (0.22–4.64)	0.06 (0.05–0.17)	0.05 (0.05–0.16)	0.05 (0.05–0.16)	**0.000**
*Biomarkers of immune activation*						
sTREM-1 pg/ml	378 (240–566)	624 (513–1154)	437 (244–1164)	348 (222–471)	337 (221–479)	**0.000**
IL-6, pg/ml	17 (6–71)	582 (36–1677)	9 (8–190)	10 (1–15)	14 (5–41)	**0.000**
sTNFR-1, pg/ml	4791 (3036–7541)	9618 (5647–19,722)	6923 (3604–8385)	4242 (2964–9908)	4445 (2794–5961)	**0.002**
CHI3L-1, ng/ml	41 (13–137)	116 (40–538)	80 (17–378)	56 (12–80)	30 (12–110)	**0.018**
IL-8, pg/ml	15 (8–39)	16 (12–51)	18 (12–34)	11 (5–21)	14 (7–42)	0.556
IP-10, pg/ml	397 (104–915)	473 (265–915)	761 (177–2096)	276 (45–916)	380 (94–874)	0.411
*Biomarkers of endothelial activation*						
Angpt-2, pg/ml	1795 (929–3396)	3181 (544–7719)	1130 (338–2887)	1728 (1120–2870)	1876 (940–2701)	0.345
sVCAM-1, ng/ml	1804 (1109–3257)	2998 (1934–4494)	1934 (1093–2248)	1549 (869–3640)	1575 (1035–2743)	**0.038**
sICAM-1, ng/ml	481 (229–880)	767 (370–933)	297 (250–1063)	165 (105–577)	481 (214–882)	0.132
Angpt-1, pg/ml	3529 (1262–9276)	3381 (944–9963)	13,389 (3103–22,903)	3157 (1622–7932)	3438 (1242–8267)	0.197
sVEGFR-1, pg/ml	183 (106–275)	233 (156–335)	172 (105–224)	83 (58–244)	183 (104–277)	0.116

Bold values indicate a significant difference between the groups

Data are median (interquartile range)

*Angpt-1* angiopoietin-1, *Angpt-2* angiopoietin-2, *CAP* community-acquired pneumonia, *CHI3L1* chitinase-3-like protein-1, *CRP* C-reactive protein, *IL-6* interleukin-6, *IL-8* interleukin-8, *IP-10* interferon-gamma-inducible protein-10, *LRTI* lower respiratory tract infection, *PCT* procalcitonin, *sICAM1* soluble intercellular adhesion molecule-1, *sTNFR-1* soluble tumor necrosis factor-1, *sTREM-1* soluble trigger receptor expressed on myeloid cells, *sVCAM-1* soluble vascular cell adhesion molecule-1, *sVEGFR1* soluble variant of vascular endothelial growth factor receptor 1

### Predictive accuracy of vital signs, clinical scores and host biomarkers for bacterial pneumonia among patients with LRTI

**Fig. 4:**
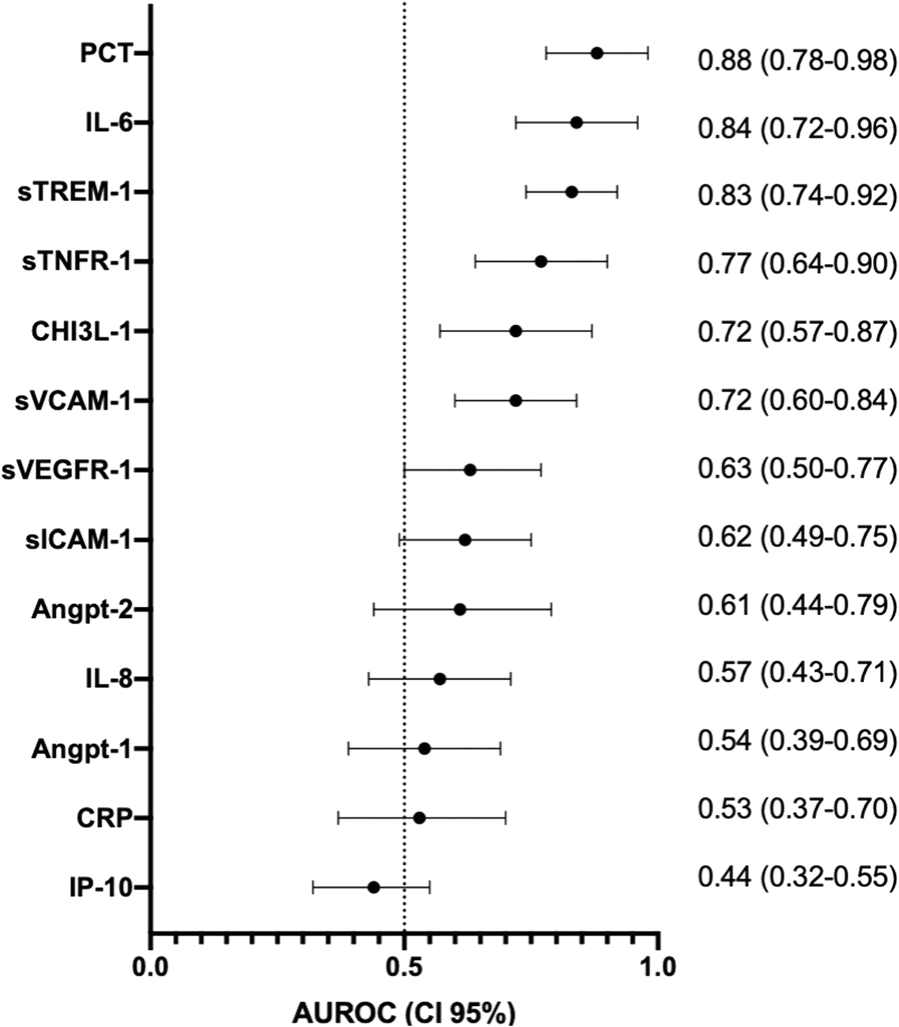
Accuracy of markers of endothelial and immune activation, measured in adults presenting with clinical lower respiratory tract infection to outpatient clinics in predicting bacterial community-acquired pneumonia. Nonparametric ROC curves were generated and AUROC were plotted to illustrate the ability of these markers to discriminate between bacterial community-acquired pneumonia and other lower respiratory tract infection. AUROCs for the outcome of each marker are presented to the right of its respective forest plot, with 95% CIs in parentheses. *Angpt-1* angiopoietin-1, *Angpt-2* angiopoietin-2, *AUROC* area under the receiver operating characteristic, *CHI3L1* chitinase-3-like protein-1, *CI* confidence interval, *CRP* C-reactive protein, *IL-6* interleukin-6, *IL-8* interleukin-8, *IP-10* interferon-gamma-inducible protein-10, *PCT* procalcitonin, *ROC* receiver operating characteristic, *sICAM-1* soluble intercellular adhesion molecule-1, *sTNFR-1* soluble tumour necrosis factor receptor-1, *sTREM-1* soluble triggering receptor expressed on myeloid cells, *sVCAM-1* soluble vascular cell adhesion molecule-1, *sVEGFR1* soluble variant of vascular endothelial growth factor receptor 1

Respiratory rate and oxygen saturation could discriminate accurately between bacterial CAP and other LRTIs (Table 3). Among biomarkers, PCT showed the best discriminating ability (AUROC 0.88; 95% CI 0.78–0.98), followed by IL-6 (0.84; 0.72–0.96) and sTREM-1 (0.83; 0.74–0.92) (Fig. 4). Of note, PCT did not perform significantly better than IL-6, sTREM-1 and sTNFR-1. Among the biomarkers of endothelial activation, sVCAM-1 was the most accurate with an acceptable ability to discriminate (AUROC 0.72; 0.60–0.84).

**Table 3:** Prognostic accuracy of vital signs and clinical scores alone and in combination with selected biomarkers (those with an area under the receiver-operating characteristic curve > 0.80) for predicting community-acquired pneumonia with a bacterial pathogen detected among those presenting with a lower respiratory tract infection at outpatient clinics in Tanzania

Model	Clinical parameter	( +) PCT	( +) IL-6	( +) sTREM1
Respiratory rate	0.82 (0.72–0.92)	0.95 (0.91–1.00)**	0.90 (0.81–0.98)*	0.89 (0.83–0.95)
Saturation	0.83 (0.75–0.91)	0.90 (0.81–0.98)	0.89 (0.81–0.96)	0.85 (0.77–0.94)
Systolic blood pressure	0.79 (0.67–0.91)	0.90 (0.82–0.98)**	0.85 (0.73–0.97)	0.86 (0.76–0.96)*
Heart rate	0.79 (0.66–0.92)	0.93 (0.88–0.99)*	0.83 (0.70–0.96)	0.86 (0.76–0.95)
CRB-65	0.76 (0.64–0.88)	0.92 (0.86–0.98)**	0.84 (0.71–0.96)*	0.86 (0.78–0.93)**
Van Vught-score	0.76 (0.66–0.87)	0.91 (0.84–0.98)**	0.85 (0.75–0.96)**	0.86 (0.78–0.94)*
PCT	–	0.88 (0.78–0.98)	0.90 (0.80–0.99)	0.89 (0.79–0.99)
IL-6	–	0.92 (0.83–1.00)	0.84 (0.72–0.96)	0.87 (0.78–0.96)
sTREM1	–	0.89 (0.79–0.99)	0.87 (0.78–0.96)	0.83 (0.74–0.92)

Data are area under the receiver-operating characteristic curve and 95% confidence interval

^*^P < 0.05, **P < 0.01, comparing the clinical parameter AUROC vs the combined clinical parameter with PCT or IL-6 AUROC

### Combination of different biomarkers and of clinical signs/scores with biomarkers

We assessed the diagnostic accuracy of varying combinations of biomarkers from similar or different pathways. We tested all biomarkers combinations (two biomarkers at a time) and none improved the diagnostic performance over using a single marker.

We also combined vital signs with the best predicting biomarkers (AUROC ≥ 0.80). The combination of respiratory rate and PCT had the highest predictive value (AUROC 0.95, 95% CI 0.91–1.00). The predictive accuracy of the combination was significantly higher than respiratory rate alone, but not than PCT alone (Table 3). The combination of systolic blood pressure, heart rate, CRB-65 and Van Vugt score with PCT also significantly improved the performance of the vital sign or score alone. We found similar results when combining vital signs and IL-6 or sTREM-1 (Table 3).

### Algorithm to predict bacterial pneumonia

**Fig. 5:**
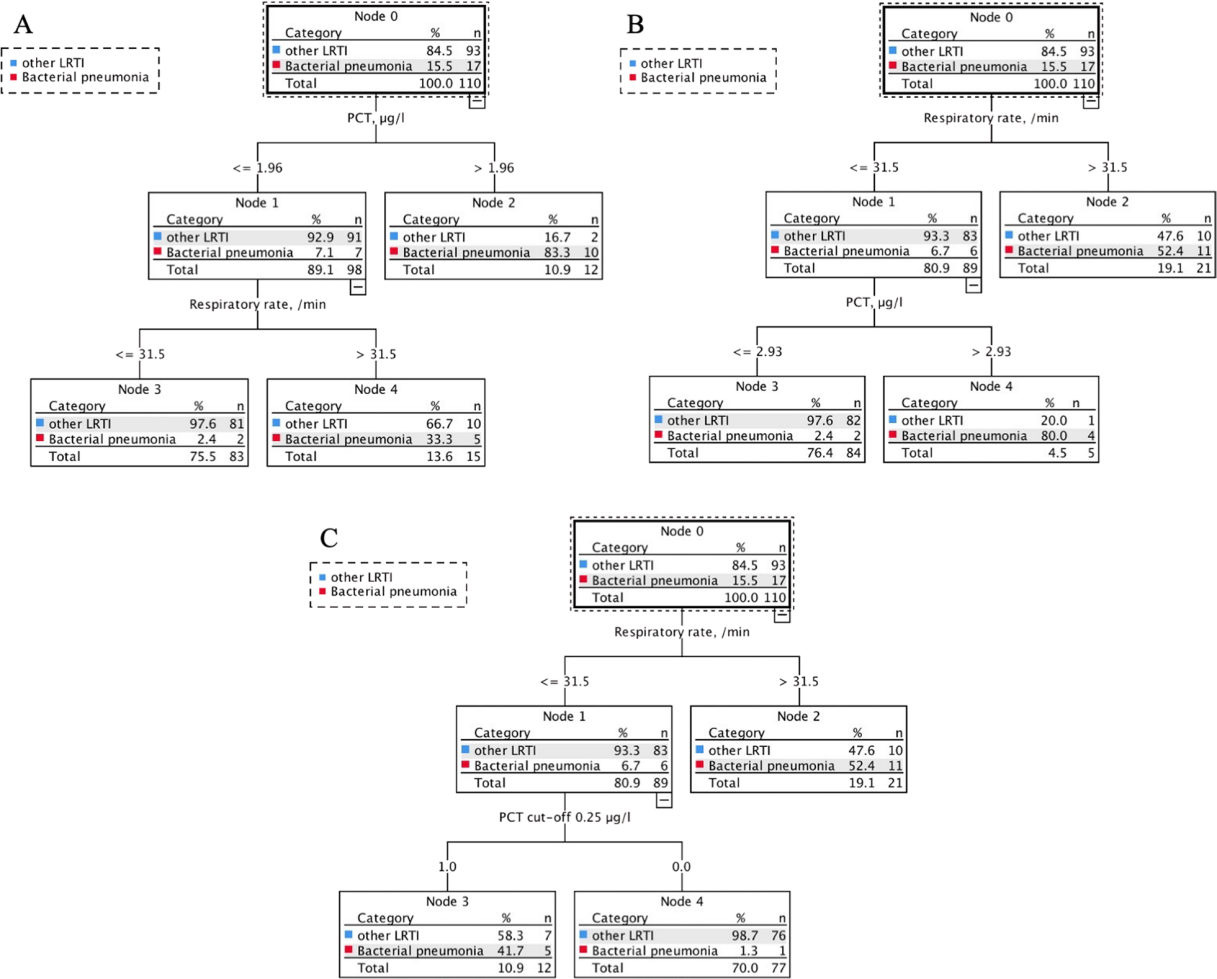
Classification and regression tree analysis to predict bacterial community-acquired pneumonia in patients presenting with lower respiratory tract infection at outpatient clinics in Tanzania. **a** All variables (vital signs and biomarkers) were added to the model. **b** Forced first respiratory rate and PCT were added to the model. **c** Forced first respiratory rate and PCT cut-off 0.25 µg/l were added to the model. For all models, the cost of misclassifying a patient that had bacterial community-acquired pneumonia as 10 times the cost of misclassifying patients that had other lower respiratory tract infection. Cut points selected by the analysis are indicated between the parent and child nodes. Below each terminal node, the predicted categorization for those patients is indicated. Algorithm performance characteristics are presented in Table 3. *PCT* procalcitonin

We performed a CRT analysis to generate algorithms to inform clinical decision making. A CRT analysis including all vital signs and biomarkers generated a classification tree including PCT first and respiratory rate second (Fig. 5A). This model had a specificity of 87% and sensitivity of 88% (Table 4). To develop an algorithm that could be used in the clinical setting and avoid unnecessary laboratory analysis (diagnostic stewardship), a CRT analysis was done forcing the respiratory rate as the first splitting variable (Fig. 5B). This model had a specificity of 88% and a sensitivity of 88%. The cut-off of PCT which was automatically selected by the model was 2.9 μg/L. To improve the sensitivity of the model and to be in line with the literature, we repeated CRT analysis with respiratory rate and a pre-defined cut-off for PCT of 0.25 μg/L (59) (Fig. 5C). This model had a sensitivity of 94% and a specificity of 82%.

**Table 4:** Performance characteristics of classification and regression tree models for predicting community-acquired pneumonia with a bacterial pathogen detected in patients presenting with a lower respiratory tract infection at outpatient clinics in Tanzania

	Prediction of community acquired pneumonia with a bacterial pathogen detected			
	Patients with a lower respiratory tract infection			Patients with radiological pneumonia
	All variables	RR ≥ 32/min and PCT ≥ 2.9	RR ≥ 32/min and PCT ≥ 0.25 µg/l	RR ≥ 32/min and PCT ≥ 0.25 µg/l
Sensitivity	88%	88%	94%	94%
Specificity	87%	88%	82%	87%
Negative likelihood ratio	0.1	0.1	0.1	0.1
Positive likelihood ratio	6.8	7.5	5.1	7.1
Negative predictive value	98%	87%	99%	93%
Positive predictive value	56%	89%	48%	89%

The identified algorithm was also tested to predict community-acquired pneumonia with a bacterial pathogen detected among patients with radiological pneumonia

*PCT* procalcitonine, *RR* respiratory rate

We further investigated whether the proposed algorithm could also be used as an additional tool to identify bacterial CAP among patients with radiological CAP. The performance remained good in this population with a similar sensitivity (94%) and specificity (87%) (Table 4).

By using this algorithm in our study population, antibiotic prescription would have been restricted to 33/110 patients (30%) instead of the 55/110 (50%) who received antibiotics, implying a drop of 40% in the absolute antibiotic prescription rate (p < 0.001). To evaluate the appropriateness of the recommendation, we compared among patients who received antibiotics, those in whom the algorithm won’t have recommended antibiotics to the other. Patients in whom antibiotics were recommended were more often HIV-infected, had a higher respiratory rate, a lower systolic blood pressure, a higher heart rate, a higher CRB-65 severity score and were more often admitted. There was no difference in mortality (Table 5). Four patients who would not have received antibiotics had sepsis, defined as a SOFA score ≥ 2 points. However, in all of them, the only sepsis criteria was a low platelets count which may not be caused by sepsis.

**Table 5:** Among all patients who received antibiotics: comparison of the characteristics of the patients classified as having a bacterial pneumonia by the algorithm (combining respiratory rate and procalcitonin) to those classified as not having a bacterial pneumonia

	Patients received antibiotics during routine care		
	Algorithm classify patients as not having a bacterial pneumonia (does not recommend antibiotics) (n = 27)	Algorithm classify patients as having a bacterial pneumonia (recommends antibiotics) (n = 28)	P value
Age, years	32 (29–42)	28 (23–36)	**0.032**
Female sex	11 (41%)	14 (50%)	0.491
HIV-1	7 (26%)	16 (57%)	**0.019**
Sepsis / septic shock^a^	4 (15%)	10 (36%)	0.121
*Symptoms and signs*			
Cough	24 (89%)	27 (96%)	0.352
Dyspnoea	7 (26%)	13 (46%)	0.114
Chest pain	8 (30%)	7 (25%)	0.700
Respiratory rate, /min	25 (23–27)	35 (26–38)	**0.000**
Abnormal auscultation	10 (37%)	7 (25%)	0.334
Saturation, %	96 (95–97)	96 (94–97)	0.129
Systolic BP, mmHg	121 (113–132)	102 (96–114)	**0.000**
Heart rate, /min	105 (87–113)	121 nn	**0.002**
*Pneumonia clinical scores*			
CRB-65 score^b^ ≥ 2	1 (4%)	9 (32%)	**0.012**
Van Vugt-score^c^, high	15 (56%)	24 (86%)	**0.019**
*Diagnosis*			
Bronchitis	17 (63%)	11 (40%)	0.079
Viral pneumonia	9 (33%)	2 (7%)	**0.020**
Bacterial pneumonia	1 (4%)	15 (54%)	**0.000**
*Management and outcome*			
Admission	4 (15%)	12 (43%)	0.037
28-day mortality	1 (4%)	2 (7%)	1.000

Bold values indicate a significant difference between the groups

Data are number (%) of patients or median (interquartile range)

^a^Sepsis/septic shock: sofa score of ≥ 2 points. ^b^CRB-65 score defined as one point for each of the following: Glasgow Coma Score < 15, respiratory rate ≥ 30/min, systolic blood pressure < 90 mmHg or diastolic blood pressure ≤ 60 mmHg, age ≥ 65. ^c^Van Vugt score was defined as one point for each of the following: absence of runny nose, presence of dyspnea, presence of crackles or diminished breath by auscultations, temperature ≥ 37.8 °C or heart rate > 100/min. High score was defined as ≥ 3 points

## Discussion

Accurate diagnostic tools to inform appropriate antibiotic use are crucial to counter the growing threat of antimicrobial resistance. In this prospective cohort of 110 adults attending outpatient clinics with a clinical LRTI in urban sub-Saharan Africa, an algorithm combining respiratory rate (cut-off of 32/min) and PCT (cut-off ≥ 0.25 μg/L) performed well to identify CAP with bacterial pathogen detected. We estimated that by applying the algorithm to the management of patients presenting with LRTI we could have reduced antibiotic use by nearly half. Furthermore, we tested the diagnostic accuracy of a panel of host response biomarkers for the identification of bacterial pneumonia, among which sTREM-1 and IL-6 displayed excellent diagnostic accuracy.

We show that PCT is a good predictor of CAP with a bacterial pathogen detected among patients with LRTIs in urban Tanzania. Our results align with a large study of patients hospitalized with CAP in the United States, which showed a strong correlation between higher PCT and an increased probability of bacterial pathogens [19] and with a large study of CAP patients hospitalized in Spain, which showed good performance of PCT to predict positive blood cultures and bacterial pathogens [20]. A systematic review evaluating the accuracy of PCT to distinguish viral from bacterial pneumonia reported limited specificity and sensitivity [21]. However, this review included a heterogeneous panel of studies, many addressing the topic of bacterial co-infection in patients with influenza. Furthermore, randomized controlled studies using PCT to guide antibiotics showed the safety of this approach [22, 23]. PCT is now available at the point-of-care which makes it suitable for implementation in daily care in sub-Saharan Africa.

By combining clinical signs and biomarkers, we show an added value compared to clinical signs alone. Furthermore, the presentation of a ready to use algorithm combining an easy-to-measure vital sign with PCT adds value since it has a high negative predictive value. In line with our findings, the combination of clinical signs and biomarkers to predict bacterial CAP added value to the clinical assessment in a Swiss cohort of LRTI patients [24].

Novel biomarkers (IL-1β, IL-6, IL-8, IL-10 and TNF) were measured in patients with CAP by Menendez et al. [20]. Their diagnostic accuracy for discriminating bacterial from viral infections was comparable to PCT, in line with our findings.

We showed a good discriminative accuracy of sTREM-1, whereas results vary in the literature. In a small cohort of patients intubated with CAP, sTREM-1 measured in the bronchoalveolar lavage was accurate to identify the presence of bacterial pneumonia [25]. In a cohort of children admitted for CAP in Italy, sTREM-1 showed a poor ability to differentiate bacterial from viral disease [26]. These discordant results might be due to the choice of antibodies utilized for the detection of sTREM-1 in the ELISAs. In line with a previous study, we also showed a good accuracy of IL-6 to identify patients with CAP and bacterial pathogen detection [20]. sTREM-1 and IL-6 also appear to be good predictors of adverse outcome in patients presenting with fever in emergency departments (including those with LRTIs and particularly those with COVID-19 [14, 27]).

This study had several limitations. First, the imperfect performance of chest X-ray in diagnosing pneumonia introduces bias in our diagnostic classification of study groups. To optimize chest X-ray interpretation and limit observer bias, two expert radiologists reviewed all images and a third expert solved discordant results. Secondly, the use of multiplex PCRs results in upper respiratory tract samples as surrogate marker of bacterial aetiology in patients with LRTI could lead to classification biases. However, a recent study from Kenya [28], showed an excellent agreement between bacteria found in upper and lower respiratory tract samples. Another study [29] showed that PCR in upper respiratory tract swabs for *S. pneumoniae* and *H. influenza* are both sensitive and specific in detecting these pathogens in adults with pneumonia. We also assumed that CAP of unknown origin were not of bacterial aetiology due to the poor yield of viral detection in nasopharyngeal swabs [7, 30]. Third, biomarker values could be affected due to co-infection with other pathogens, such as malaria or dengue. To minimize the impact of co-infection, we excluded patients with documented bacterial infections presenting with respiratory tract symptoms, such as typhoid fever or bacteremia of a source other than the respiratory tract. Although we are aware that infections like malaria might affect the biomarkers value, we kept these patients as it represents the reality in this setting [31–33]. Fourth, as there is no perfect screening test, we may have missed some tuberculosis diagnoses in our study population. However, we used an inclusive tuberculosis definition to minimise underdiagnosis. Finally, the sample size of our study population is limited and our results were not confirmed in an external cohort. If the developed algorithm is validated in another population, its impact should to be tested in an intervention trial.

## Conclusions

Here we provide new data supporting a simplified algorithm combining an easy-to-measure vital sign, respiratory rate, with a biomarker easily measurable at the point-of-care, PCT, that displayed an excellent predictive accuracy in identifying adult CAP patients with documented bacterial infection. Findings from our study support the potential use of biomarkers for antibiotic stewardship and management of patients with LRTIs in sub-Saharan Africa. However, our results need confirmation in larger cohorts of patients with lower respiratory tract infections.

## Supplementary Information

**Additional file 1: Table S1.** Presence of bacteria in nasopharyngeal swabs. Comparison of patients with community-acquired pneumonia and a control group of patients with dengue without any respiratory symptoms. **Table S2.** Baseline characteristics, vital signs, and outcome of patients with community-acquired pneumonia and documented viral pathogen versus patients with community-acquired pneumonia of unknown origin. **Table S3.** Baseline characteristics, vital signs, management, and outcome of enrolled patients. Comparison between patients with community-acquired pneumonia and documented bacterial pathogen versus patients with other lower respiratory tract infections.

## Data Availability

The datasets used and/or analysed during the current study are available from the corresponding author on reasonable request.

## Abbreviations

AMR: Antimicrobial resistance
Angpt-1: Angiopoietin-1
Angpt-2: Angiopoietin-2
AUROC: Area under the receiver operating characteristic curve
CAP: Community acquired pneumonia
CRP: C-reactive protein
IL-6: Interleukin-6
LRTI: Lower respiratory tract infection
PCR: Polymerase chain reaction
PCT: Procalcitonin
qSOFA: Quick Sequential Organ Failure Assessment
STREM-1: Soluble triggering receptor expressed by myeloid cells-1
