# Supplementary material for: Clinical sign and biomarker-based algorithm to identify bacterial pneumonia among outpatients with lower respiratory tract infection in Tanzania
Source: BMC Infect Dis. 2022 Jan 6;22:39. doi: 10.1186/s12879-021-06994-9 (PMC8735728; doi:10.1186/s12879-021-06994-9)
Supplement: Supplementary file 1 — Additional file 1: Table S1. Presence of bacteria in nasopharyngeal swabs. Comparison of patients with community-acquired pneumonia and a control group of patients with dengue without any respiratory symptoms. Table S2. Baseline characteristics, vital signs, and outcome of patients with community-acquired pneumonia and documented viral pathogen versus patients with community-acquired pneumonia of unknown origin. Table S3. Baseline characteristics, vital signs, management, and outcome of enrolled patients. Comparison between patients with community-acquired pneumonia and documented bacterial pathogen versus patients with other lower respiratory tract infections. [file 12879_2021_6994_MOESM1_ESM.docx]

**Additional tables**

Table S1: Presence of bacteria in nasopharyngeal swabs. Comparison of patients with community-acquired pneumonia and a control group of patients with dengue without any respiratory symptoms.

|  | Community-acquired pneumonia (n=32) | Dengue without respiratory symptoms (n=101) | P value |
| --- | --- | --- | --- |
| *Streptococcus pneumoniae* | 15 (47) | 12 (12) | **0.000** |
| *Haemophilus influenzae* | 3 (9) | 1 (1) | **0.043** |
| *Moraxella catarrhalis* | 6 (19) | 6 (6) | **0.028** |
| *Staphylococcus aureus* | 5 (16) | 19 (19) | 0.683 |
| *Legionella pneumophila* | 0 (0) | 0 (0) | - |
| *Mycoplasma pneumoniae* | 0 (0) | 0 (0) | - |
| *Chlamydia pneumoniae* | 0 (0) | 0 (0) | - |

Data are number (%) of patients

Table S2: Baseline characteristics, vital signs, and outcome of patients with community-acquired pneumonia and documented viral pathogen versus patients with community-acquired pneumonia of unknown origin.

|  | Viral community-acquired pneumonia (n=8) | Community-acquired pneumonia of unknown origin (n=7) | P value |
| --- | --- | --- | --- |
| Age, years | 31 (29-51) | 33 (32-64) | 0.451 |
| Female sex | 3 (38) | 2 (29) | 1.000 |
| HIV-1infection | 4 (50) | 1 (14) | 0.282 |
| Sepsis / septic shock* | 1 (13) | 1 (14) | 1.000 |
| Symptoms and signs | | | |
| Respiratory rate, /min | 26 (25-34) | 24 (23-26) | 0.102 |
| Saturation, % | 96 (94-96) | 97 (96-98) | 0.059 |
| Systolic blood pressure, mmHg | 119 (109-123) | 126 (120-132) | **0.037** |
| Heart rate, /min | 115 (104-120) | 98 (82-108) | 0.132 |
| Outcome | | | |
| 28-day mortality | 1 (13) | 0 (0) | 1.000 |

Data are number (%) of patients or median (interquartile range).

* Sepsis or septic shock defined as a sofa score of ≥2 points.

Table S3: Baseline characteristics, vital signs, management, and outcome of enrolled patients. Comparison between patients with community-acquired pneumonia and documented bacterial pathogen versus patients with other lower respiratory tract infections.

|  | All (n=110) | Bacterial community-acquired pneumonia (n=17) | Other lower respiratory tract infections (n=93) | P value |
| --- | --- | --- | --- | --- |
| Age, years | 29 (23-39) | 28 (24-35) | 30 (23-40) | 0.640 |
| Female sex | 58 (53) | 8 (47) | 50 (54) | 0.611 |
| HIV-1infection | 36 (33) | 13 (76) | 23 (25) | **<0.001** |
| Co-infection | 22 (20) | 2 (12) | 20 (22) | 0.516 |
| - Malaria | 7 (6) | 1 (6) | 6 (6) | 1.000 |
| - Dengue | 7 (6) | 0 (0) | 7 (7) | 0.593 |
| - Other | 9 (8) | 1 (6) ^◊^ | 8 (9) ^±^ | 1.000 |
| Sepsis / septic shock* | 27 (25) | 5 (29) | 22 (24) | 0.612 |
| Symptoms and signs | | | | |
| Cough | 102 (93) | 17 (100) | 86 (92) | 0.593 |
| Dyspnoea | 27 (25) | 6 (35) | 21 (23) | 0.263 |
| Chest pain, | 25 (23) | 4 (24) | 21 (23) | 1.000 |
| Respiratory rate, /min | 25 (23-29) | 34 (26-37) | 24 (22-26) | **<0.001** |
| Abnormal auscultation | 29 (26) | 8 (47) | 21 (23) | **0.035** |
| Saturation, % | 97 (96-98) | 95 (93-96) | 97 (96-98) | **<0.001** |
| Systolic blood pressure, mmHg | 117 (104-123) | 100 (97-107) | 120 (107-125) | **<0.001** |
| Heart rate, /min | 108 (92-122) | 129 (117-138) | 106 (90-115) | **<0.001** |
| Pneumonia clinical scores | | | | |
| CRB-65 score^#^ ≥ 2 | 12 (12) | 5 (29) | 7 (8) | **0.012** |
| Van Vugt-score°, high | 66 (60) | 16 (94) | 50 (54) | **0.002** |
| Management and outcome | | | | |
| Admission | 20 (19) | 7 (41) | 13 (16) | **0.008** |
| Antibiotic prescription | 55 (50) | 16 (94) | 39 (42) | **<0.001** |
| 28-day mortality | 6 (5) | 2 (12) | 4 (4) | 0.232 |

Data are number (%) of patients or median (interquartile range).

Bacterial community-acquired pneumonia: community-acquired pneumonia with a bacterial aetiology detected; Other lower respiratory tract infections include community-acquired pneumonia with viral or unkown orgin and bronchitis.

1 adenovirus gastro-enteritis. ± 1 gastro-enteritis of unknown origin, 2 West nile virus, 2 rickettsioses, 3 urinary tract infections (E. coli). * Sepsis or septic shock defined as a sofa score of ≥ 2 points. # CRB-65 score defined as one point for each of the following: Glasgow Coma Score < 15, respiratory rate ≥ 30/min, systolic blood pressure < 90 mmHg or diastolic blood pressure ≤ 60 mmHg, age ≥ 65. ° Van Vugt score was defined as one point for each of the following: absence of runny nose, presence of dyspnea, presence of crackles or diminished breath by auscultations, temperature ≥ 37.8 °C or heart rate >100/minute. High score was defined as ≥ 3 points.
